# Photocatalytic Activity of Boron-Modified SnO_2_ Nanoparticles for Crystal Violet Removal

**DOI:** 10.3390/nano16171101

**Published:** 2026-09-01

**Authors:** Daniela Negoescu, Anca Vasile, Oana Mocioiu, Crina Anastasescu, Mihaela Gherendi, Daniela C. Culita, Irina Atkinson, Simona Petrescu, Cristian Hornoiu, Veronica Bratan

**Affiliations:** 1 Institute of Physical Chemistry–Ilie Murgulescu of the Romanian Academy, 060021 Bucharest, Romania; danielan@icf.ro (D.N.); avasile@icf.ro (A.V.); omocioiu@icf.ro (O.M.); canastasescu@icf.ro (C.A.); dculita@icf.ro (D.C.C.); iatkinson@icf.ro (I.A.); spetrescu@icf.ro (S.P.); chornoiu@icf.ro (C.H.); 2 National Institute for Lasers, Plasma and Radiation Physics, 077125 Magurele, Romania; mihaela.gherendi@inflpr.ro

**Keywords:** SnO_2_ nanoparticles, boron, doped semiconductors, cationic dye, photodegradation

## Abstract

Boron (B)-modified SnO_2_ samples with various B concentrations (1, 2, and 5 at%) were successfully synthesized using the sol–gel method. The effect of the B/SnO_2_ molar ratio on the crystal structure, microstructure, optical, and photocatalytic properties was investigated. The samples were characterized by X-ray diffraction (XRD), N_2_ adsorption–desorption experiments, Fourier transform infrared (FTIR) spectroscopy, X-ray photoelectron spectroscopy (XPS), Diffuse reflectance UV–Vis (DR UV–Vis) and photoluminescence (PL) spectroscopy. A decrease in particle size was observed with increasing B concentration. The B-doped samples exhibited a higher fraction of microporosity and a larger specific surface area than those of undoped SnO_2_. FTIR spectra display characteristic absorption of B species. The band gap values were lower than that of bulk SnO_2_, and the PL results indicated a reduced electron–hole recombination rate upon boron doping. The presence of defects, such as oxygen vacancies, is highlighted. The nanoparticles exhibited excellent photocatalytic activity toward the degradation of crystal violet (CV) dye, achieving a removal efficiency under UV irradiation of over 90% for the 5 at% B-doped SnO_2_ sample after 90 min.

## 1. Introduction

Nowadays, rapid industrialization generates significant amounts of by-products, which pose serious environmental challenges. The discharge of industrial effluents containing toxic contaminants adversely affects the physical, chemical, and biological quality of aquatic ecosystems, highlighting the need for efficient and environmentally friendly treatment technologies [1,2,3,4].

Among the available approaches, heterogeneous photocatalysis has emerged as a particularly promising technique due to its simplicity, low operating costs, and ability to degrade organic pollutants under mild conditions [5,6,7,8,9]. This technology relies on the generation of electron–hole pairs upon light irradiation of semiconductor materials. The photogenerated charge carriers react with water and dissolved oxygen to produce reactive oxygen species (ROS), which are responsible for the degradation and even the mineralization of organic contaminants. Owing to their tunable electronic structure, high chemical stability, and versatility, metal oxide semiconductors have attracted considerable attention as photocatalysts for environmental remediation. Furthermore, reducing their size to the nanocrystalline scale endows them with special properties, compared with their bulk counterparts, such as a high surface-to-volume ratio [10].

Tin dioxide (SnO_2_) is an n-type semiconductor characterized by low toxicity, natural abundance, high chemical stability, and excellent photochemical resistance [11,12,13]. SnO_2_-based materials have been extensively studied for a wide range of applications, including oxidation catalysis [14,15,16], photocatalysis [17,18,19], solar cells [20,21], and gas sensing [22,23]. Nevertheless, despite its strong oxidation capability, resulting from its relatively low valence-band potential, its photocatalytic efficiency is hindered by its wide band gap (~3.6 eV), low specific surface area, and rapid recombination of photogenerated charge carriers [7,24,25]. Consequently, considerable research efforts have focused on improving its photocatalytic efficiency, including control of size and morphology, defect structure engineering, elemental doping, and the synthesis of composite materials [24,26,27,28].

Numerous studies have demonstrated that doping significantly improves structural, optical, and photocatalytic properties of SnO_2_ [28,29,30,31,32]. A relatively new doping strategy, the incorporation of boron into a metal oxide crystal lattice, can lead to modifications in the structure including changes in unit cell geometry, variations in bond lengths, or the formation of new electronic levels that could hinder the electron–hole recombination and improve the light absorption [33,34,35,36,37,38,39].

Boron-doped SnO_2_ has been investigated for various applications, including dye degradation, lithium-ion battery electrodes, gas sensing, and optoelectronic devices. Kumar and collaborators [40] have synthesized boron-doped SnO_2_ nanoparticles, which have proven active in photocatalytic hydrogen generation by water splitting and in degradation of dyes, due to the generation of defects by doping. Filippatos et.al. [41] have made a comparative study and examined the impact of B and In doping on the structural, electrical, and optical properties of SnO_2_. They have investigated the effect of dopant in the interstitial/substitutional site on the modification of band gap energy and were able to determine the benefits of using these compounds in photocatalytic applications. Kong et al. [42] synthesized boron-doped films and evaluated them in the degradation of organic pollutants with excellent results under visible light irradiation. Doping resulted in a strong visible-light response and a lower electron–hole recombination rate. Nevertheless, to date, the number of research articles on the influence of boron doping on SnO_2_ properties and its application in photocatalysis remains limited.

Therefore, the objective of the present work is to investigate the photocatalytic performance of several boron-doped SnO_2_ samples synthesized via a sol–gel approach. By studying a relatively underexplored photocatalytic system, this research aims to contribute to the understanding of the influence of boron incorporation on the physicochemical properties of SnO_2_ and their relationship with photocatalytic activity.

## 2. Materials and Methods

### 2.1. Synthesis of the Materials

SnO_2_-based materials were prepared using a simple sol–gel method. Tin (II) chloride dihydrate (SnCl_2_·2H_2_O, Carl Roth, Germany) and boric acid (H_3_BO_3_, Chimreactiv, Romania) were used as precursors. Appropriate amounts of boric acid corresponding to boron concentrations of 1, 2, and 5 at% were dissolved together with 3 g of SnCl_2_·2H_2_O in 50 mL of distilled water under continuous magnetic stirring for 1 h. Subsequently, a 4 M NaOH solution was added dropwise until the pH = 10. The resulting mixture was further stirred for 2 h and then aged at 27 °C for 24 h. The obtained gel was dried at 80 °C for 20 h and subsequently calcined in air at 500 °C for 3 h, using a heating rate of 5 °C/min. Finally, the resulting powder was washed with distilled water to remove residual sodium chloride, and dried again at 80 °C.

### 2.2. Characterization of the Samples

X-Ray diffraction patterns of the catalysts were obtained utilizing a Rigaku diffractometer, model Ultima IV, from Tokyo, Japan, operating in parallel-beam geometry with Cu-K_α_ (λ = 1.5406 Å) radiation across a 2θ range of 10–80°. The scan rate was set at 5°/min with a step size of 0.02°.

The textural properties of the samples, including the specific surface area (S_BET_) and pore characteristics, were determined by nitrogen adsorption–desorption measurements at −196 °C using a Micromeritics ASAP 2020 automated gas sorption analyzer (Norcross, GA, USA). Prior to the measurements, the samples were degassed under vacuum at 200 °C for 4 h to remove adsorbed impurities.

The surface morphology of synthesized samples was analyzed, without any conductive coating, using a scanning electron microscope, SEM Quanta 3D FEG instrument (FEI, Brno, Czech Republic).

FTIR spectra were recorded with a JASCO FT/IR-4700 spectrometer (JASCO Corporation, Hachioji, Japan), spectral range: 4000–400 cm^−1^, resolution 0.4 cm^−1^. Nanopowders were immobilized in KBr pellets.

Surface analysis using X-ray photoelectron spectroscopy (XPS) was performed using a Thermo Fisher ESCALAB 250Xi+ instrument (Waltham, MA, USA) equipped with thin anodes (Al and Ag). A monochromatized Al Kα X-ray source with an excitation energy of 1486.6 eV and a spot diameter of 900 μm was used. The pressure in the analysis chamber was maintained in the range of 10^−7^–10^−6^ Pa. The analyzer was operated at a constant analyzer energy (CAE) of 200 eV, and the energy step for each acquisition was 0.1 eV.

The UV–vis absorption characteristics of sample nanoparticles were studied by a Perkin Elmer Lambda 35 UV–vis spectrophotometer, Shelton, CT, USA, in the range 900–200 nm, using spectralon as a white reference.

The photoluminescence spectra were registered using a Carry Eclipse spectrophotometer (Agilent Technologies, Santa Clara, CA, USA) with excitation/emission slits set at 10/10. Measurements were performed at an excitation wavelength of λ_ex_ = 300 nm for 0.005 g powder suspended in ultrapure water, at room temperature.

### 2.3. Photocatalytic Tests

The photocatalytic performance of the synthesized materials was evaluated by studying the degradation of crystal violet dye under UV irradiation. The experimental setup has been previously reported and successfully employed by our group for the evaluation of various photocatalytic materials [6,9]. The experiments were carried out at room temperature using quartz microreactors (inner diameter: 21.5 mm; height: 37.2 mm). 10 mL of an aqueous solution of CV (2 × 10^−5^ M) containing 5 or 2 mg of photocatalyst (corresponding to 500 mg/L, and 200 mg/L catalyst loading) was magnetically stirred in the dark for 30 min, and then irradiated with a 254 nm UV light (UV-VL-215c lamp, with 1.53 × 10^15^ photons/sec/cm^2^). At predetermined irradiation times, 1 mL aliquots were withdrawn, centrifuged, and analyzed using a PerkinElmer Lambda 35 UV–Vis spectrophotometer (Shelton, CT, USA) by monitoring the characteristic absorption at λ = 590 nm. The dye removal efficiency (RE%) was calculated according to the equation:

RE = [(C_0_ − C)/C_0_]·100 (1)

where C_0_ represents the initial concentration of the dye solution, and C is the concentration of the dye solution at irradiation time t. All photocatalytic experiments were performed in triplicate at a dye concentration of 500 mg/L and in duplicate at 200 mg/L. The results are presented as mean ± standard deviation (SD).

### 2.4. Radical Scavenging Experiments

To identify the reactive species involved in the photocatalytic process, radical scavenging experiments were performed by adding 0.1 mmol of selected scavengers to the reaction solution before irradiation. Potassium iodide (KI), silver nitrate (AgNO_3_), ethanol, and *p*-benzoquinone (p-BQ) were used as scavengers for photogenerated holes (h^+^), electrons (e^−^), hydroxyl radicals (•OH), and superoxide radicals (•O_2_^−^), respectively. All experiments were carried out under the same conditions as the photocatalytic degradation tests.

### 2.5. Monitoring of Hydroxyl Radical (•OH) Photogeneration

The photogeneration of (•OH) radicals was monitored with a 10 mM coumarin (Merck, Darmstadt, Germany) solution and 0.005 g suspended catalyst exposed to simulated solar irradiation AM 1.5 (Peccel solar simulator, Peccell Technologies, Yokohama, Japan) for 0, 5, 10, and 20 min. The photogenerated (•OH) radicals at the catalyst surface react with coumarin solution, leading to umbelliferone formation, a fluorescent compound monitored with a Carry Eclipse fluorescence spectrometer from Agilent Technologies, Santa Clara, CA, USA (λ_em_ = 451 nm), slits set to 10 nm in excitation and emission, λ_exc_ = 330 nm.

## 3. Results

**Figure 1:**
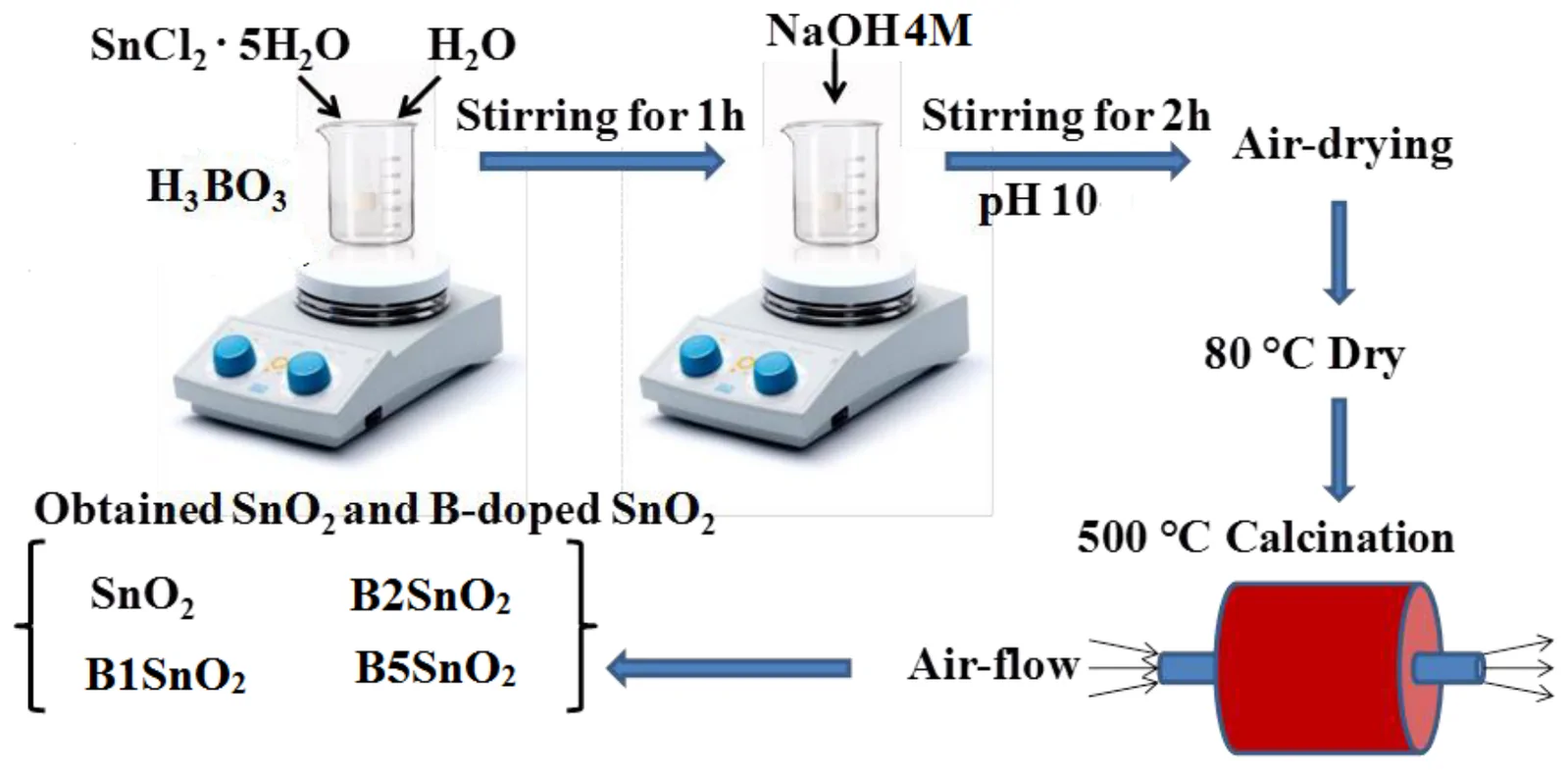
Synthesis of materials powders by the sol–gel method.

Schematic representations of the samples’ synthesis are shown in Figure 1. Four samples were prepared: pure SnO_2_ and B-doped SnO_2_ with boron atomic concentrations of 1, 2, and 5 at%, designated as SnO_2_, B1SnO_2_, B2SnO_2_, and B5SnO_2_, respectively.

### 3.1. Structural, Textural, and Morphological Properties

**Figure 2:**
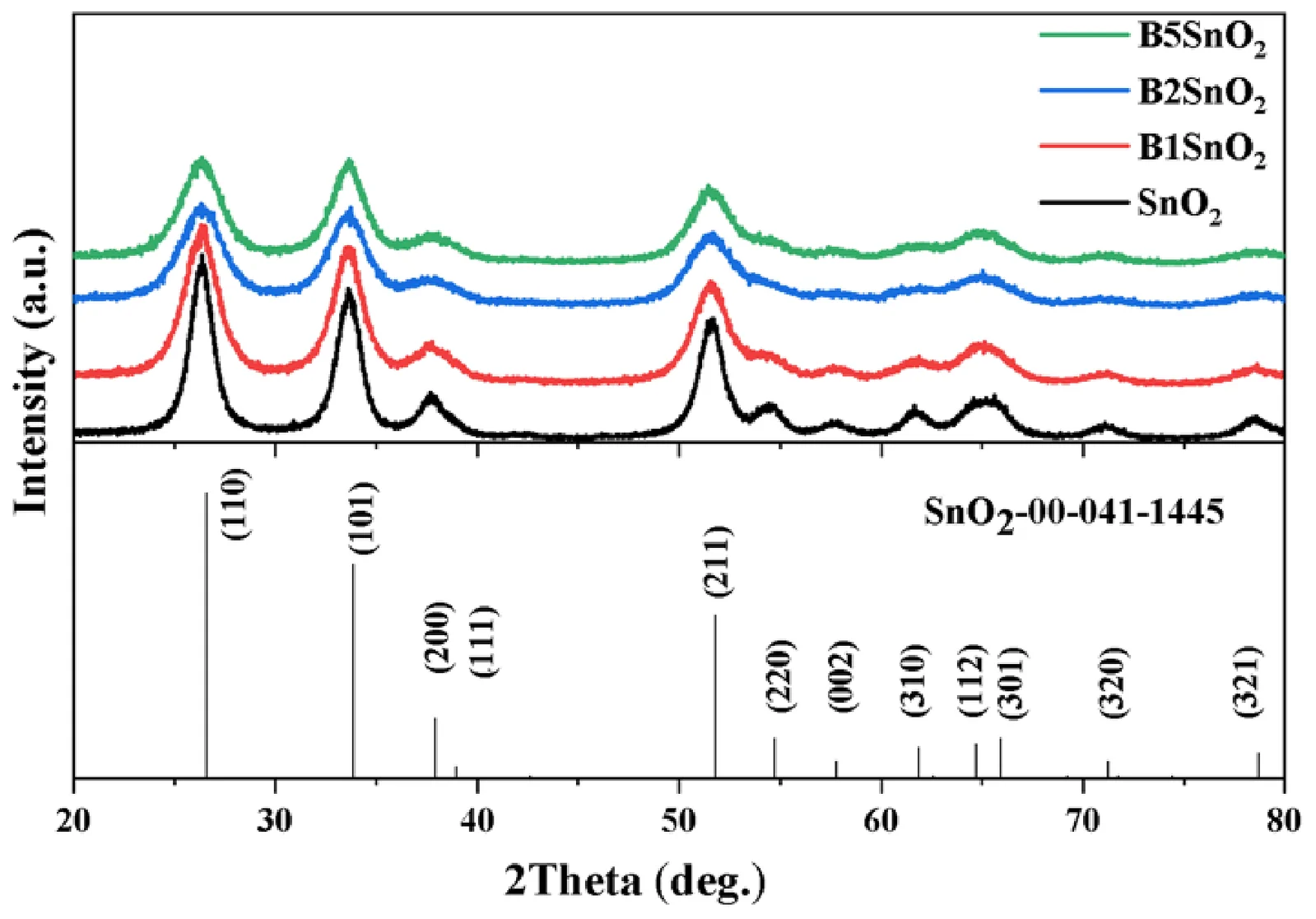
XRD patterns of pure and B-doped SnO_2_ nanoparticles.

Figure 2 displays the XRD patterns of pure and boron-doped SnO_2_ nanoparticles. All identified reflections can be indexed to the tetragonal cassiterite phase of SnO_2_ (space group P4_2_/mnm), in agreement with JCPDS Card No. 41-1445. The diffraction peaks located at ~26.3°, 33.6°, 37.6°, 51.5°, 54.5°, 57.7°, 61.6°, 65.9°, 71.2°, and 78.1° correspond to the (110), (101), (200), (211), (220), (002), (310), (301), (320), and (321) crystallographic planes, respectively. The most intense reflections are associated with the (110), (101), and (211) planes. No secondary phases or impurity-related peaks were detected. That means that either the boron concentration is below the XRD detection limit or that it is successfully incorporated into the SnO_2_ lattice. A gradual decrease in diffraction peak intensity accompanied by slight peak shifts was observed upon boron doping (Table 1), suggesting modifications of the crystal lattice. As the B content increases, the intensity of XRD peaks decreases, indicating a slight decrease in the crystallinity of the nanoparticles.

The lattice parameters and unit cell volume were calculated for all samples, and the obtained values are presented in Table 1. Compared with the reference values reported for bulk cassiterite SnO_2_ (a = b = 4.738 Å, c = 3.187 Å, V = 71.54 Å^3^), all the samples exhibit slightly expanded lattice parameters and unit-cell volumes, which may indicate the presence of intrinsic lattice defects. According to Porte et al. [43] interstitial defects and doubly charged oxygen vacancies tend to expand the lattice, whereas neutral oxygen vacancies generally lead to lattice contraction. As shown in Table 1, the B1SnO_2_ and B2SnO_2_ samples exhibit larger unit-cell volumes than unmodified SnO_2_, suggesting the presence of a higher concentration of defects and/or interstitial boron species. Interstitial boron is expected to increase the lattice volume, whereas substitutional incorporation should lead to lattice contraction as the ionic radius of B^3+^ (0.023 nm) is significantly smaller than that of Sn^4+^ (0.071 nm). The subsequent decrease in the unit-cell volume (V_cell_), particularly observed for B5SnO_2_, suggests that a fraction of the boron ions is incorporated into substitutional sites within the cassiterite structure.

The average crystallite size was estimated using the Williamson–Hall method, and the corresponding values are also listed in Table 1. All samples consist of nanocrystalline SnO_2_ with the crystallite size progressively decreasing from 4.9 nm for SnO_2_ to 3.0 nm for the B5SnO_2_ sample. This trend suggests that doping inhibits crystallite growth during synthesis. The simultaneous reduction in crystallite size and modification of the lattice parameters provide further evidence for the successful incorporation of boron into the SnO_2_ structure, most likely through interstitial occupancy.

**Figure 3:**
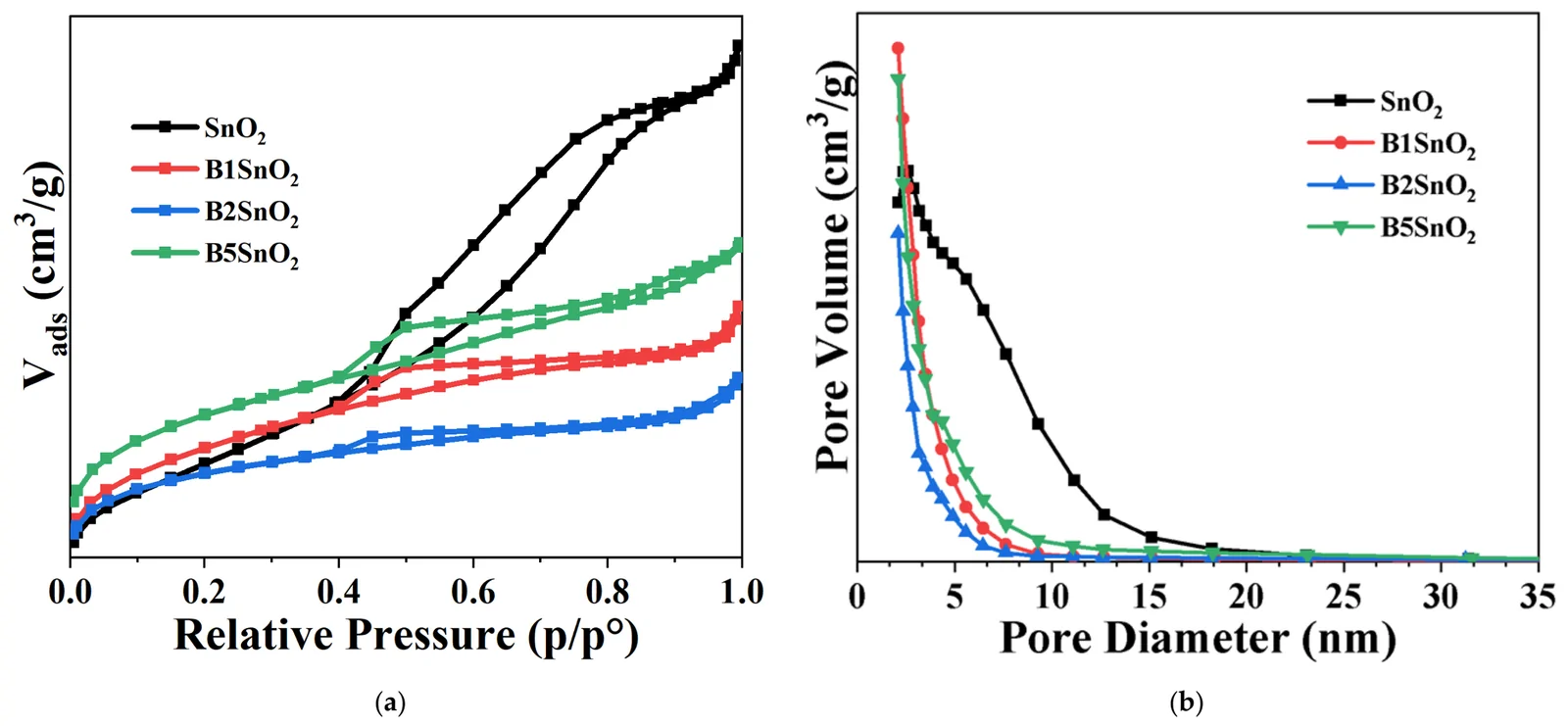
Comparative (**a**) nitrogen adsorption–desorption isotherms. (**b**) Pore size distributions of nO_2_ and B-modified SnO_2_ materials.

To investigate the influence of boron incorporation on the textural properties of the synthesized materials, nitrogen adsorption–desorption measurements were performed, and the corresponding isotherms are presented in Figure 3a. According to the IUPAC classification [44] all samples exhibit a typical type IVa profile with an H3/H4 hysteresis loop in the relative pressure range of 0.4–1.0. Pure SnO_2_ sample shows a higher N_2_ uptake and a broader hysteresis loop than the boron-doped samples, suggesting a larger mesopore volume and a wider pore size distribution. Upon boron incorporation, the hysteresis loop becomes narrower, indicating the development of a more uniform slit-like pore structure accompanied by a reduction in mesopore volume. It can be observed that all boron-doped samples have a significant microporous contribution. These observations are further supported by the pore-size distribution curves presented in Figure 3b.

The textural parameters (summarized in Table 2) reveal a complex evolution of the porosity upon boron incorporation. Although the BET specific surface area does not increase monotonically with boron concentration, the B5SnO_2_ sample exhibits the highest surface area among all investigated materials. This behavior suggests that boron incorporation affects the porous structure through competing mechanisms rather than a single concentration-dependent effect. It is worth noting the significant increase in the microporous surface area (S_micro_) with boron doping. The undoped sample has a negligible microporous contribution of only 2.3 m^2^/g, while B1SnO_2_, B2SnO_2_ and B5SnO_2_ show S_micro_ values of 19.1, 33.0, and 32.2 m^2^/g, respectively, representing an increase of over an order in magnitude. This progressive development of microporosity indicates a significant influence of doping on the porous texture of the material, probably by modifying the aggregation behavior of nanoparticles or by generating micropores within the SnO_2_ matrix. Conversely, the total pore volume decreases substantially by doping. The average pore diameter similarly decreases from 5.40 nm for SnO_2_ to 3.97–4.41 nm for the doped samples, confirming the narrowing of the pore size distribution. These observations are consistent with the evolution of the hysteresis loop character from H3 to H4 with increasing boron content, as the microporosity increases.

**Figure 4:**
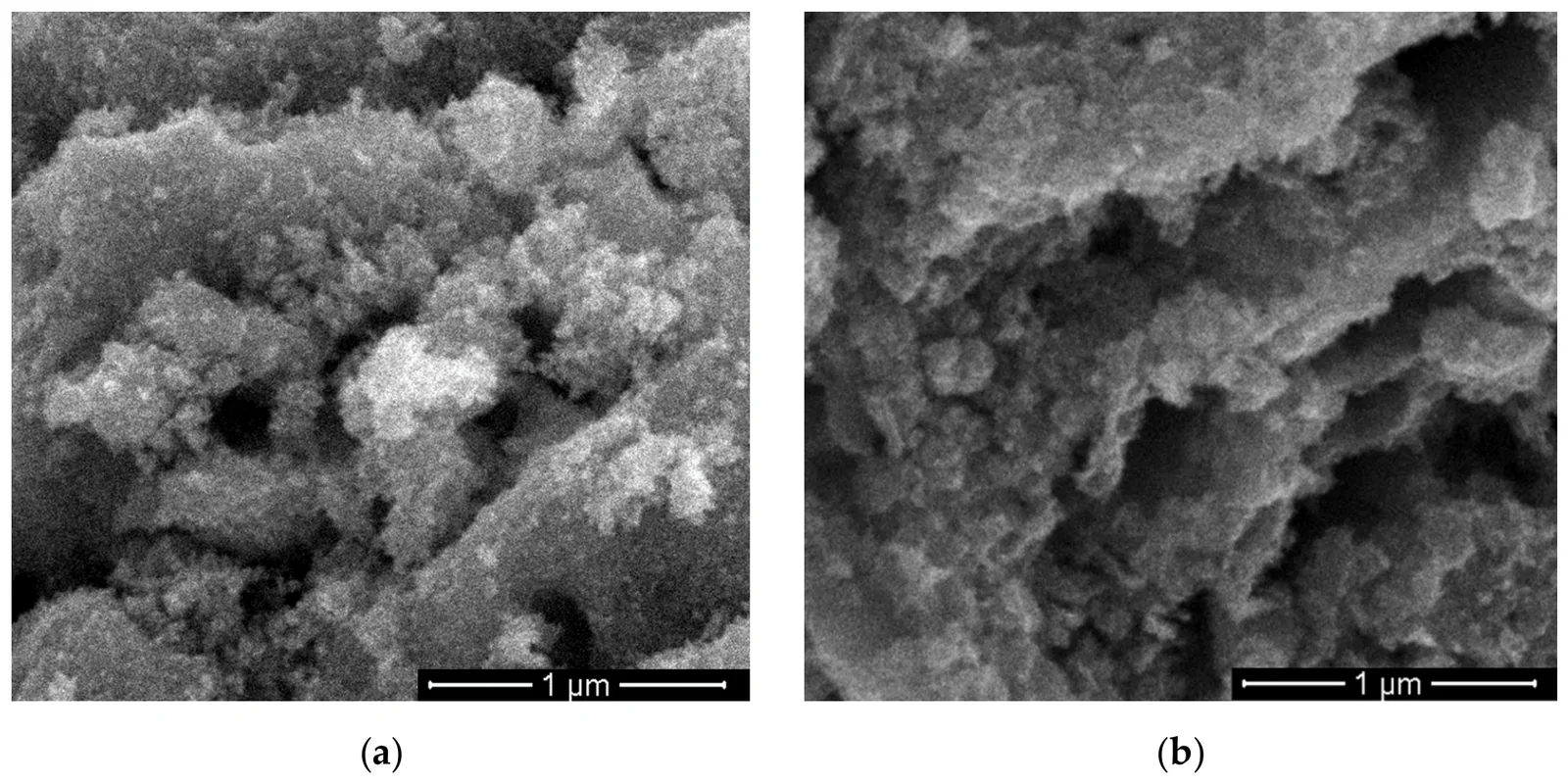
SEM micrographs of (**a**) pristine SnO_2_; (**b**) B2SnO_2_.

The SEM micrographs, illustrated in Figure 4, revealed that the morphology consists of agglomerated nanoparticle networks, characteristic of oxide nanomaterials synthesized by the sol–gel method. The B2SnO_2_ sample displays smaller and better-defined particles compared to the undoped sample which supports the XRD observation that boron integration induces structural defects and limits long-range crystal growth.

**Figure 5:**
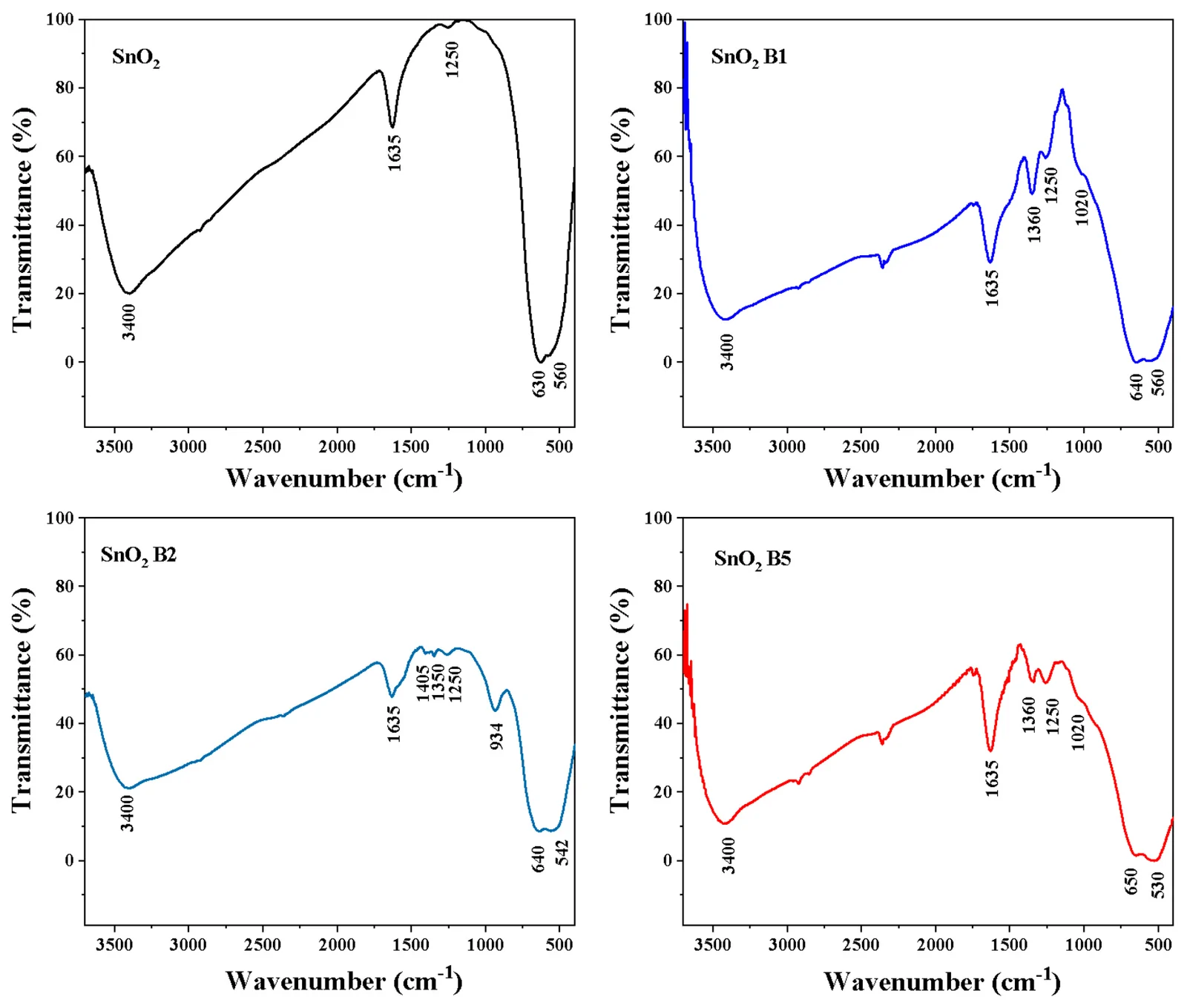
FTIR spectra of synthesized samples.

FTIR spectroscopy was employed to investigate the functional groups present in the synthesized samples. The recorded spectra are presented in Figure 5. The broad absorption band centered around 3400 cm^−1^ together with the weaker band at 1635 cm^−1^ are assigned to the stretching and bending vibrations of surface hydroxyl groups and adsorbed water molecules respectively [45].

The characteristic vibrational features of the SnO_2_ lattice are observed in the low-wavenumber region of the spectra. Two distinct absorption bands located at approximately 630–650 cm^−1^ and 530–560 cm^−1^ (depending on boron concentration) are attributed to Sn-O stretching vibrations within the cassiterite structure and to O-Sn-O vibrations, respectively [46,47]. Slight variations in the position, intensity, and width of these bands are observed after boron incorporation. Such spectral changes are commonly associated with reduced crystallite size, lattice distortion, and the generation of structural defects. In particular, the broadening of the absorption bands in the boron-doped samples suggests modifications of the local bonding environment induced by dopant [46]. Considering the small ionic radius of boron, the observed shift in the O-Sn-O bending vibration (with the following order of frequency: SnO_2_ = B1SnO_2_ > B2SnO_2_ > B5SnO_2_) is consistent with the preferential incorporation of boron into interstitial positions, which locally distorts the SnO_2_ lattice and modifies the bond strength. However, some boron atoms may occupy tin sites, causing a shift in the Sn-O stretching vibrations frequency as it is observed especially for B5SnO_2_ sample (650 cm^−1^ compared with 630 cm^−1^ for SnO_2_). This shift suggests the insertion of boron at substitutional site in accordance with XRD data [47]. The small band located at 1250 cm^−1^ is associated with Sn-OH vibrations [40,45].

In addition to the lattice- and hydroxyl-related bands described above, the doped samples exhibit distinct absorption features in the 900–1500 cm^−1^ region and provide further evidence for the successful incorporation of boron species. Stretching vibrations associated with BO_3_ groups appear in the 1150–1600 cm^−1^ range, while BO_4_ tetrahedral borate groups exhibit characteristic absorption bands in the 800–1150 cm^−1^ range [48]. Accordingly, the bands observed at 928, 1020 and 1100 cm^−1^ are attributed to BO_4_ species [35,40], whereas the band located near 1365 cm^−1^ is assigned to the asymmetric stretching vibrations of B-O bods in trigonal BO_3_ units [48,49]. Contributions from Sn–O–B linkages cannot be excluded [49].

### 3.2. Determination of Surface Chemical States and Optical Properties

**Figure 6:**
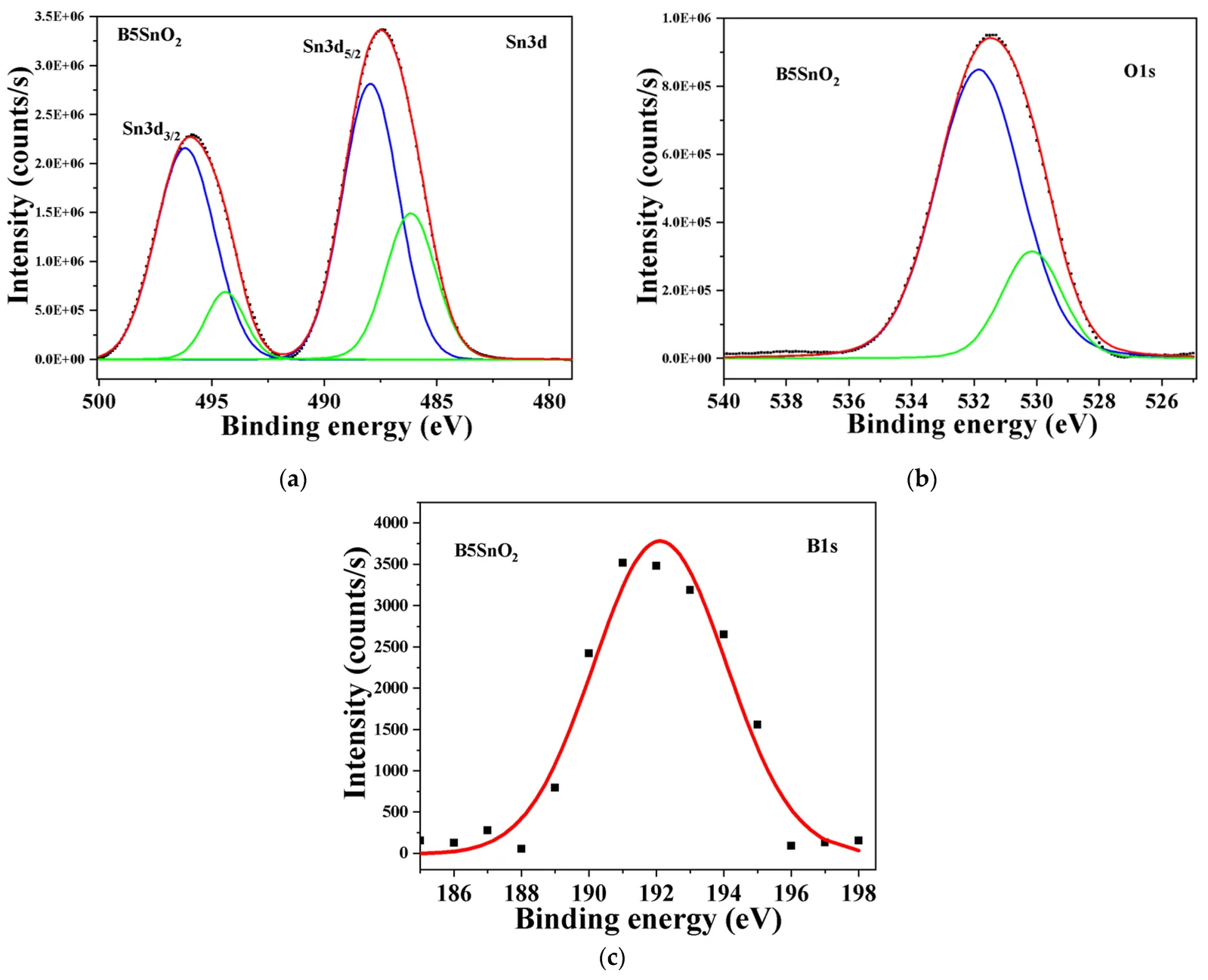
XPS deconvolution for the B5SnO_2_ sample of (**a**) Sn3d, (**b**) O1s and (**c**) B1s.

The surface chemical composition of the samples and oxidation states of the constituent elements were investigated by X-ray photoelectron spectroscopy (XPS). Figure 6 presents the high-resolution XPS spectra of the Sn 3d and O 1s regions for the B5-SnO_2_ sample, which is representative of the investigated materials. The corresponding spectra for the remaining samples are provided in the Supplementary Material (Figures S1–S3).

The deconvolution of the Sn 3d spectra indicates the presence of tin in two oxidation states with distinct chemical environments: Sn^4+^, corresponding to the dominant SnO_2_ phase, and a minor Sn^2+^ component. The Sn 3d_5_/_2_ peak assigned to Sn^4+^ appears at a slightly higher binding energy than the value reported for stoichiometric SnO_2_ (486.4 eV) [50], suggesting the presence of structural defects even in the undoped sample. The secondary oxidation state identified by XPS is most likely associated with Sn atoms located in grain-boundary regions, where structural disorder is more pronounced. The presence of these Sn^2+^ species has been correlated with the formation of oxygen vacancies, as previously reported in the literature [51].

This interpretation is further supported by the O 1s spectra, which can be deconvoluted into two components corresponding to different oxygen chemical environments. The peak centered at approximately 530 eV is assigned to lattice oxygen (O^2−^), whereas the higher-binding-energy component, located at approximately 531–532 eV, is commonly attributed to oxygen species associated with oxygen vacancies or defect-related surface oxygen [51].

Only a weak B 1s signal is detected at approximately 190–192 eV, even for the sample containing 5 at% boron. The low intensity of this signal indicates that boron is present in relatively low concentration at the particle surface, suggesting that a significant fraction of the dopant is incorporated into the SnO_2_ lattice rather than segregated into a separate boron-rich phase. Furthermore, the binding energy of the B 1s peak is considerably higher than that of elemental boron (187 eV), indicating that boron is chemically bonded to oxygen within the oxide framework. This assignment is consistent with the formation of B–O bonds and possible Sn–O–B linkages, in agreement with the FTIR results [52,53].

The binding energy values, intensity, and atomic compositions for all samples are inserted in Supplementary Material, Table S1a–d.

**Figure 7:**
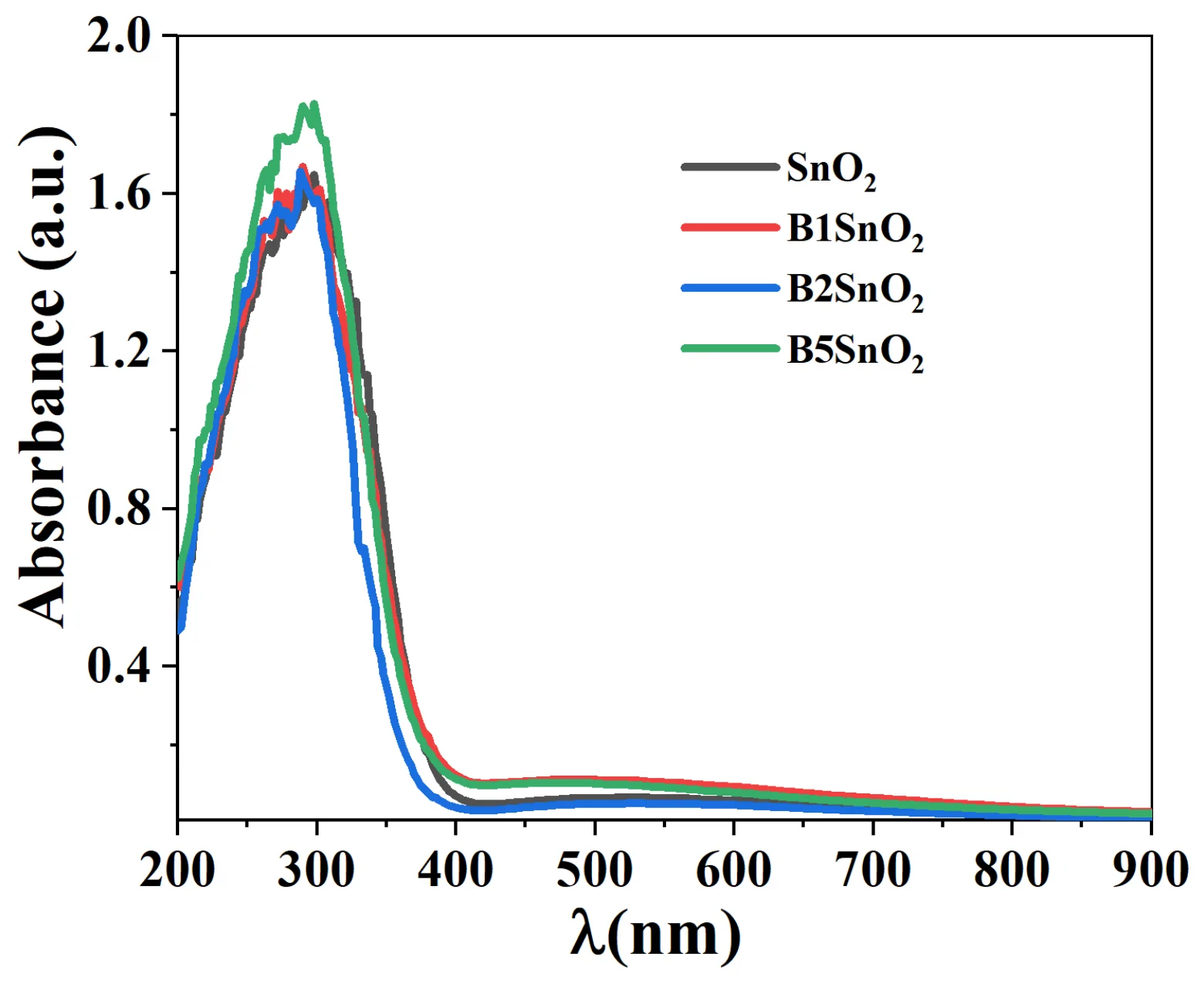
DR UV-Vis spectra of SnO_2_ and boron-modified SnO_2_.

The light absorption properties determine the potential applicability of a material as a photocatalyst. Therefore, the UV–Vis spectra were recorded and are presented in Figure 7. All samples exhibit strong absorption in the UV range (200–400 nm), originating from band-to-band transitions in SnO_2_ [54,55,56]. Additionally, a slight absorption is present in the visible range, with higher intensity for the B1Sn and B5Sn samples, which may be attributed to electronic transitions involving defect levels within the band gap [55,57]. By analogy with MoO_3_ oxide, this weak absorption band can also be due to a localized surface plasmon resonance (LSPR). The oxygen vacancies introduce new electronic levels right below the Fermi level and increase the free charge carrier concentration, leading to the excitation of LSPR [58].

The optical bandgap energies were estimated using Tauc’s method, which differentiates between the natures of the electronic transitions. For this purpose, a plot of [F(R)/hν]^1/η^ versus hν is represented, where hν is the photon energy and η is a parameter that depends on the type of the electronic transition. It takes the value of 1/2 for direct transitions and 2 for indirect transitions. The band gap values were calculated for both direct and indirect transitions (Figures S4 and S5, Supplementary Material), and the results are presented in Table 3. The obtained direct band gap values (3.35–3.48 eV) are lower than that of bulk SnO_2_ (3.6 eV), while remaining relatively similar among the investigated samples. The lowest value was obtained for B1SnO_2_, whereas the highest was observed for B2SnO_2_. The same trend is preserved for the indirect band gap, although the corresponding values are considerably lower (2.92–3.13 eV).

The decrease in the band gap relative to bulk SnO_2_ (3.6 eV) is most likely attributed to the higher defect density, which introduces additional electronic states within the band gap [51], in accordance with XRD, XPS and FTIR results. However, the observed differences in the band-gap values among the samples may result from the combined effects of lattice strain, quantum size effects (where E_g_ increases with decreasing particle size), and variations in the defect structure, including boron-related defects. Additionally, interstitial boron may act as a shallow donor, increasing the free-electron concentration in the SnO_2_ lattice. An increase in carrier concentration can shift the apparent optical band gap toward higher energies through the Burstein–Moss effect. In contrast, substitutional boron is expected to promote the formation of oxygen vacancies for charge compensation, leading to a reduction in the band-gap energy. Therefore, the experimental band-gap values suggest that interstitial incorporation is favored at lower boron concentrations (B1SnO_2_ and B2SnO_2_), whereas both interstitial and substitutional boron species may coexist in the B5SnO_2_ sample [59].

**Figure 8:**
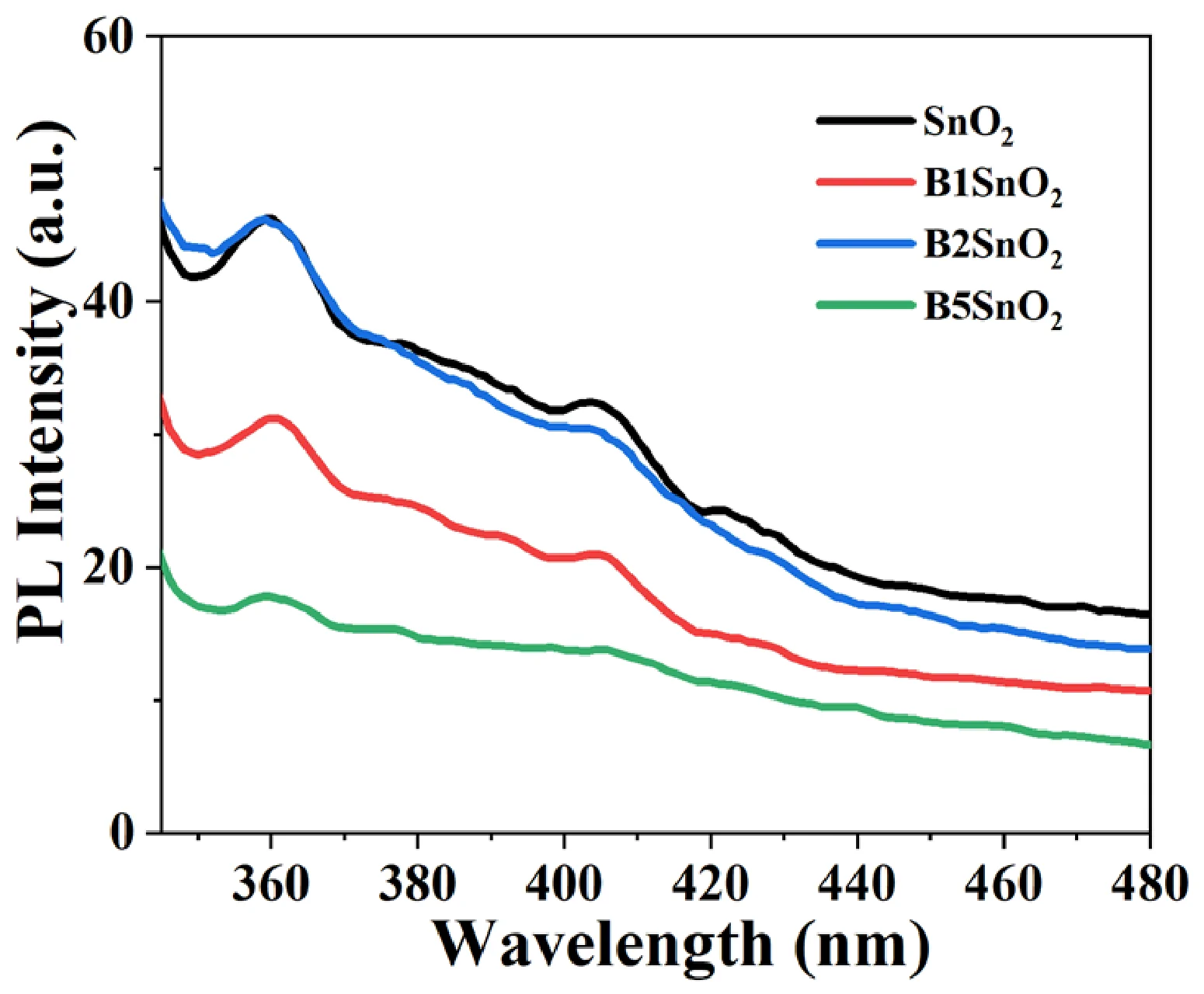
Comparative PL emission spectra of bare and boron-modified (experimental conditions: 0.005 g powder suspended in 3 mL of water, at room temperature).

PL spectra are depicted in Figure 8. When excited with 300 nm light, the SnO_2_-based samples emphasized two emission peaks located around 358 and 403 nm, respectively. Figure 8 also shows a decrease in radiative recombination with the addition of boron, suggesting better use of the photogenerated charges in the photocatalytic processes.

### 3.3. Photocatalytic Experiments

The photocatalytic activity of the synthesized samples was evaluated through the degradation of crystal violet (CV) under UV irradiation. CV, a cationic dye with toxic, mutagenic, and carcinogenic effects, was selected as a model pollutant for degradation studies because it is extensively used in industrial sectors, such as the textile and pharmaceutical industries [4,60,61]. Consequently, its presence in wastewater is expected, underscoring the need for development of efficient remediation strategies.

**Figure 9:**
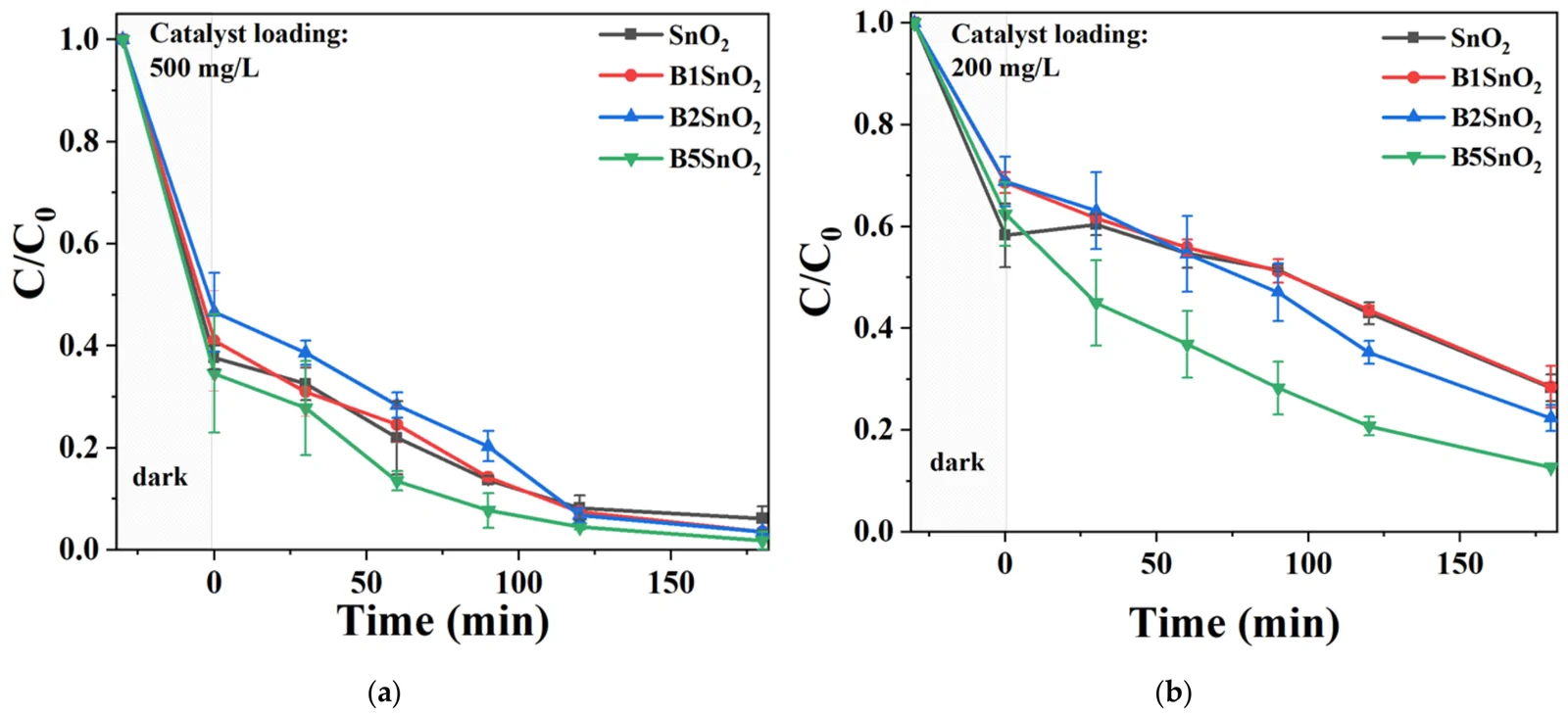
UV photodegradation of CV dye (initial concentration: 2 × 10^−5^ M) in the presence of the synthesized samples at two different catalyst loadings: (**a**) 500 mg/L and (**b**) 200 mg/L. Data are presented as mean ± SD. Error bars represent the standard deviation of three independent experiments for (**a**) and two independent experiments for (**b**).

The experiments were conducted at pH 5.5, corresponding to the natural pH of the reaction medium. Prior to irradiation, the suspensions were magnetically stirred in the dark for 30 min to establish adsorption–desorption equilibrium between the photocatalyst particles, dye molecules, and dissolved oxygen in the solution. The evolution of CV normalized concentration (C/C_0_) as a function of irradiation time at two different catalyst loadings is presented in Figure 9.

A considerable amount of CV was adsorbed onto the surface of all the investigated materials, with the corresponding adsorption values for 500 mg/L catalyst loading listed in Table 4. This behavior can be attributed to the cationic nature of the dye, arising from the positively charged nitrogen atom located at the end of one of its three molecular branches [60]. The point of zero charge (pzc) of SnO_2_ is typically reported in the range of 3.5–4.0 [62]. Since the working pH in this study (5.5) is higher than the pzc, the surface of the SnO_2_ nanoparticles is expected to be negatively charged (due to the hydroxyl groups on the surface). This favors the electrostatic interaction with positively charged crystal violet molecules, enhancing their adsorption onto the catalyst surface. The formation of adsorbed dye species has also been reported previously in the literature [60] and was further supported by the violet coloration of the photocatalyst powders observed after the dark adsorption step.

Upon UV irradiation, all samples exhibited good photocatalytic performance, with the B5SnO_2_ sample achieving almost complete decolorization of the dye after 90 min at a catalyst loading of 500 mg/L (Figure 9a). However, the relatively high conversion achieved at this catalyst loading resulted in smaller differences in photocatalytic efficiency among the samples. Therefore, additional experiments were performed at a lower catalyst loading of 200 mg/L, while maintaining the same reactor configuration and experimental conditions (Figure 9b). The trend in activity was preserved, with SnO_2_ and B1SnO_2_ exhibiting very similar photocatalytic behavior, whereas increasing the boron content resulted in progressively enhancement of CV degradation, with B5SnO_2_ again showing the highest conversion.

**Figure 10:**
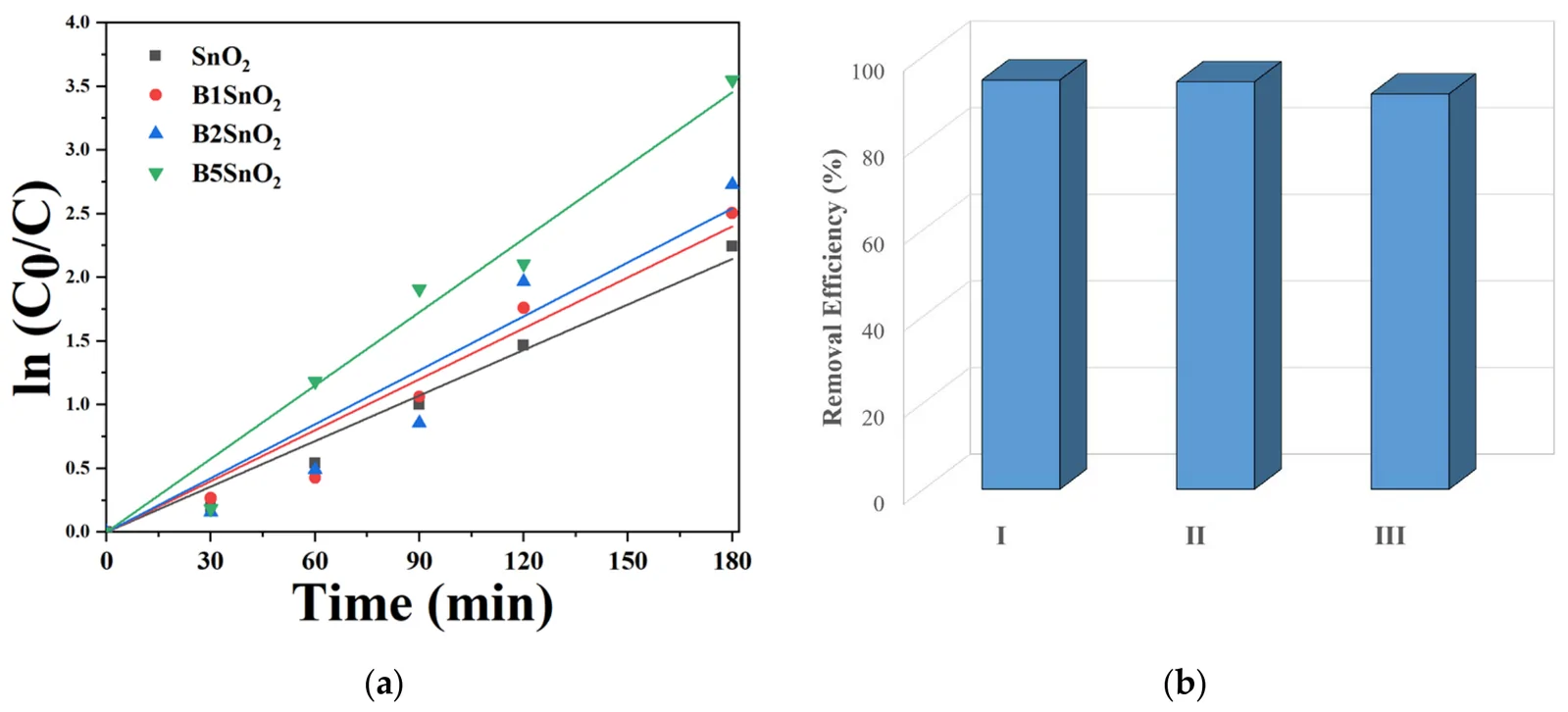
(**a**) Pseudo-first-order kinetic analysis of CV photodegradation over SnO_2_ and B-doped SnO_2_ samples; (**b**) reusability experiments (3 consecutive cycles: I, II, III). Experimental conditions: catalyst loading, 500 mg/L; initial CV concentration, 2 × 10^−5^ M; irradiation time, 3 h.

The photocatalytic degradation kinetics were analyzed using the Langmuir–Hinshelwood model, assuming pseudo-first-order reaction kinetics [6]. The corresponding kinetic plots are presented in Figure 10, while the apparent rate constants (k) and half-life values (t_1_/_2_), included to facilitate comparison among the photocatalysts, are summarized in Table 3. The apparent degradation rate constant was determined from the slope of the linear plot of ln(C_0_/C) versus irradiation time, where C_0_ is the CV concentration before irradiation, and C is the concentration at time t. The kinetic analysis revealed that the boron-doped samples exhibited higher apparent degradation rate constants than unmodified SnO_2_ confirming the beneficial effect of boron incorporation. Additionally, the apparent rate constant increased progressively with increasing boron concentration.

The reusability of the most active photocatalyst (B5SnO_2_) was evaluated over three consecutive photocatalytic degradation cycles, each lasting 180 min. After each cycle, the photocatalyst was recovered by centrifugation, thoroughly washed with distilled water to remove residual reaction products, and dried overnight at 70 °C before being reused in the subsequent experiment. As shown in Figure 10b, the photocatalytic removal efficiency remained high throughout the three consecutive cycles, with only a slight decrease after repeated use. These results demonstrate the good stability and reusability of the B5SnO_2_ photocatalyst, indicating that boron incorporation does not adversely affect the structural integrity or photocatalytic performance of the material under the investigated reaction conditions.

**Figure 11:**
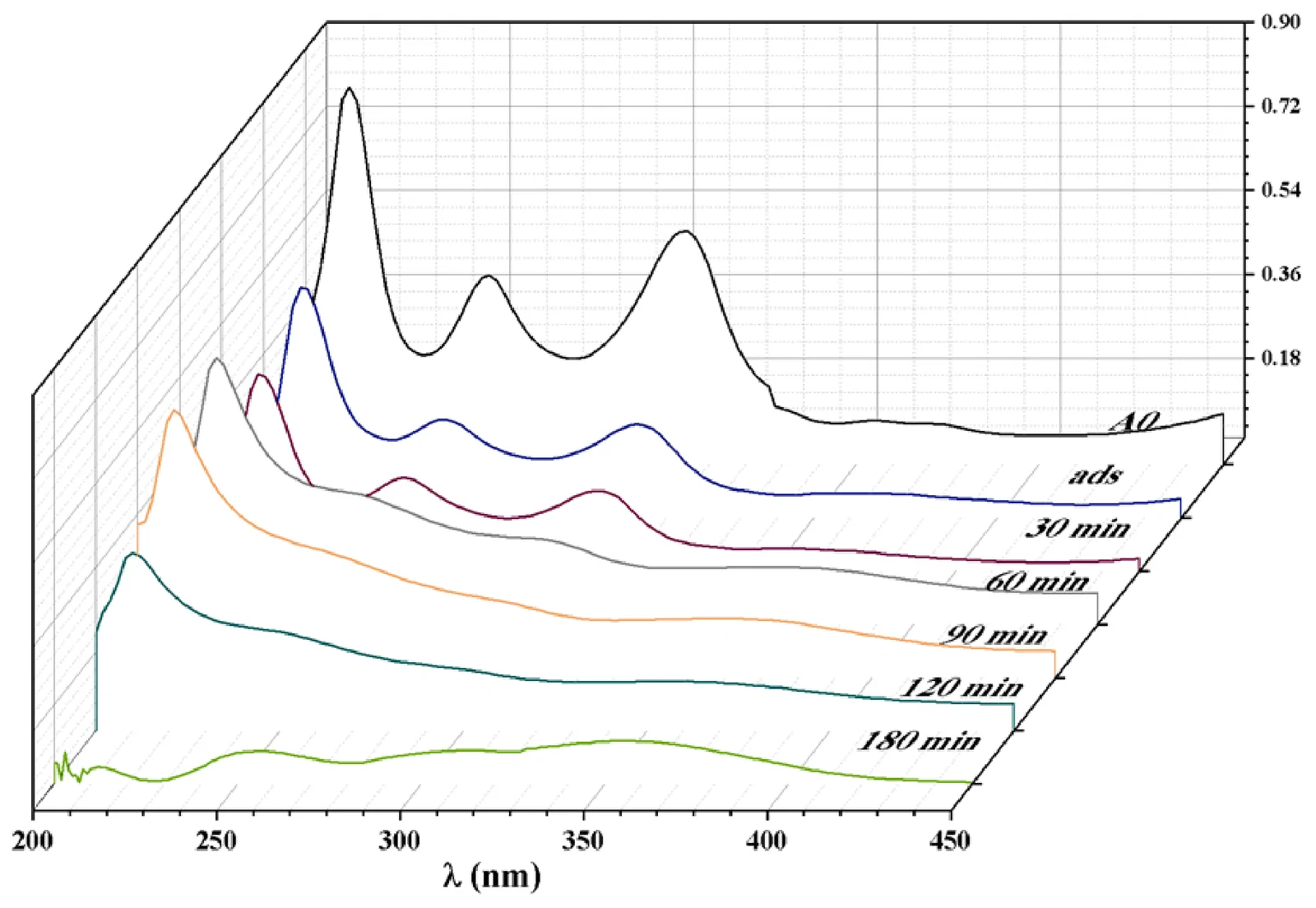
Time-evolution of CV absorbance during photodegradation over B5SnO_2_ sample in the wavelength range 200–450 nm. Experimental conditions: catalyst loading, 500 mg/L; initial CV concentration, 2 × 10^−5^ M.

To obtain additional information regarding the mineralization of crystal violet, the evolution of the solution absorbance in the UV region was monitored throughout the photocatalytic reaction (Figure 11). UV absorbance at 254 nm (UV254) is widely used as an indicator for dissolved organic carbon (DOC) [63]. A pronounced decrease in the UV254 was observed during the photocatalytic reaction, consistent with a progressive mineralization of crystal violet. Nevertheless, the persistence of several absorption features in the UV region suggests that complete mineralization was not achieved within the investigated reaction time and that organic intermediates were still present in solution.

## 4. Discussion

The high photocatalytic performance of all samples toward CV degradation and the differences observed between the doped and undoped materials can be explained by structural modification induced by synthesis conditions.

XRD analysis revealed that boron is incorporated predominantly at interstitial sites, whereas in the B5SnO_2_ sample a fraction of the boron ions may occupy substitutional sites within the cassiterite structure. Furthermore, the increase in microporous surface area, together with the higher BET surface area observed at high boron doping suggests that boron plays an active role in restructuring the porous network of SnO_2_ with potential implications for surface-dependent properties such as heterogeneous catalysis, as catalytic performance is closely related to the number of accessible active sites.

All photocatalysts exhibited high CV adsorption capacities owing to the strong electrostatic attraction between the negatively charged SnO_2_ surface and the cationic dye molecules. Enhanced adsorption generally promotes photocatalytic activity by increasing the availability of pollutant molecules at the active sites [64]. However, adsorption alone cannot account for the observed photocatalytic performance, as pure SnO_2_ exhibited a higher adsorption capacity than the B5SnO_2_ sample, yet showed lower overall removal efficiency.

The photocatalytic degradation of organic dyes generally proceeds through the formation of reactive oxygen species, primarily superoxide radicals (•O_2_^−^) and hydroxyl radicals (•OH). To gain further insight into the photocatalytic mechanism, the positions of the conduction band (CB) and valence band (VB) relative to the normal hydrogen electrode (NHE) were estimated using Equations (2) and (3).

E_VB_ = X - E_e_ + 0.5E_g_ (2)

E_CB_ = E_VB_ - E_g_ (3)

where X is the absolute electronegativity of SnO_2_ (6.24 eV), E_e_ is the energy of free electrons on the hydrogen scale (4.5 eV), and E_g_ is the band gap energy determined from diffuse reflectance UV–Vis spectroscopy [54]. The calculated band edge potentials are summarized in Table 3.

In addition, the ability of the samples to generate hydroxyl radicals under light irradiation was evaluated using the coumarin fluorescence probe method. The photoluminescence peak located at 451 nm (λ_ex_ =330 nm; λ_em_ = 451 nm) indicates the formation of the umbelliferone, a photoluminescent product resulting from the interaction of coumarin with the photogenerated hydroxyl radicals under AM 1.5 irradiation.

**Figure 12:**
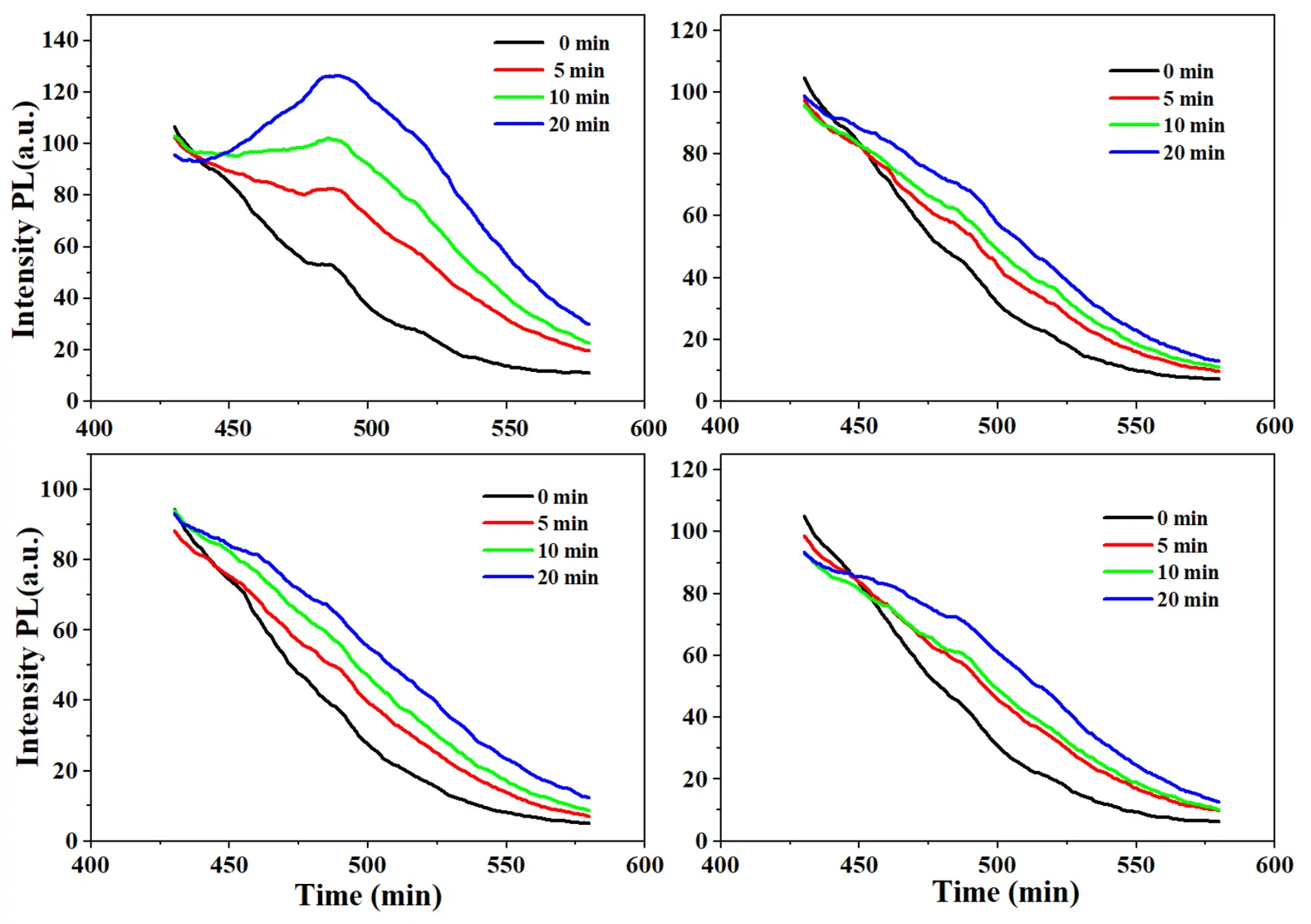
Comparative monitoring of hydroxyl radical generation under simulated solar irradiation over the investigated catalysts.

As shown in Figure 12, only pure SnO_2_ exhibited a distinct emission peak at 451 nm after 20 min of irradiation, indicating the generation of hydroxyl radicals. In contrast, boron incorporation markedly suppressed •OH formation. Comparison with the reference TiO_2_ P25 Degussa photocatalyst (Supplementary Material, Figure S6) further reveals that both pure and boron-doped SnO_2_ samples generate significantly lower amounts of hydroxyl radicals under identical experimental conditions.

Although the calculated VB positions (+3.42 to +3.56 V vs. NHE) indicate that the photogenerated holes would possess sufficient oxidation potential to oxidize surface water or hydroxyl groups, thereby generating hydroxyl radicals (•OH), the experimental results do not support this prediction. A possible explanation is related to the high concentration of structural defects identified by XRD, FTIR, and XPS. Previous studies have suggested that oxygen vacancies may locally modify the electronic structure of SnO_2_, resulting in an effective valence-band edge that is less positive than the standard •OH/H_2_O oxidation potential. Under these conditions, hydroxyl radical generation was suppressed [54]. Since all synthesized samples including undoped SnO_2_, exhibit defect-rich structures, this mechanism may explain the generally low •OH production observed in the present study.

**Figure 13:**
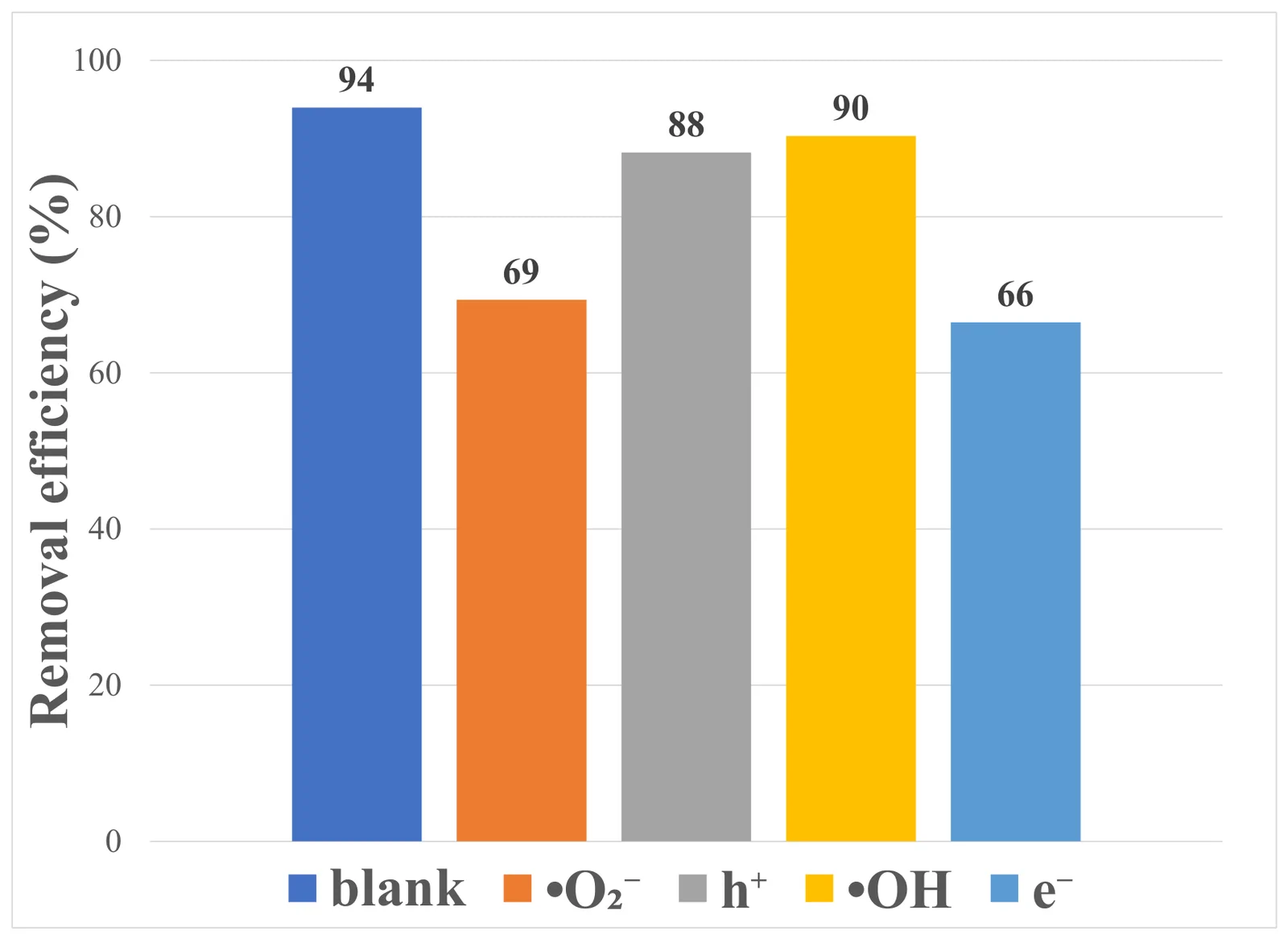
CV photodegradation over B5SnO_2_ catalyst conducted in the presence of different scavengers.

To identify the main active species responsible with CV photodegradation, experiments in the presence of electrons (e^−^), superoxide radical anions (•O_2_^−^), hydroxyl radicals (•OH) and holes (h^+^) scavengers were carried out for B5SnO_2_ sample (500 mg/L catalyst loading; 2 × 10^−5^ M CV initial concentration). As shown in Figure 13, the CV removal efficiency is decreased by the presence of the •O_2_^−^ and e^−^ scavengers, indicating that these species play a major role in the degradation process. In contrast, hydroxyl radical scavengers produced a much smaller effect, in agreement with the coumarin fluorescence experiments.

The results indicate that photodegradation takes place via reactive oxygen species formed in solution. Electrons from the conduction band of the catalyst reduce dissolved molecular oxygen and formed •O_2_^−^. Superoxide anion plays a dual role: it directly oxidizes CV molecules and cascades into •OH formation via •HO_2_/H_2_O_2_ intermediates [65], which further degrade the pollutant. The generated reactive oxygen species attack the crystal violet molecules through successive N-demethylation, cleavage of the triphenylmethane structure, and ring-opening oxidation reactions, ultimately leading to progressive mineralization into CO_2_ and H_2_O. Nevertheless, alternative degradation pathways including direct oxidation by photogenerated holes and/or dye sensitization cannot be excluded.

However, the photocatalytic performance of the synthesized materials is comparable to the best results reported in the literature [6,66,67,68,69] for the degradation of crystal violet (Table S2, Supplementary Materials). The obtained results suggested that the photocatalytic activity is probably associated with the electronic states associated with oxygen vacancies, which may directly provide electrons for the reduction in adsorbed molecular oxygen to superoxide radicals (•O_2_^−^). The reduced electron–hole recombination observed for the boron-doped samples, as evidenced by the photoluminescence results, is expected to increase the lifetime of photogenerated charge carriers, thereby enhancing the probability of electronic transfer.

## 5. Conclusions

In summary, SnO_2_ nanoparticles doped with different boron concentrations (1, 2, and 5 at%) were successfully synthesized using a simple sol–gel method. XRD analysis confirmed that all samples crystallized in the cassiterite structure and revealed a gradual decrease in crystallite size with increasing boron content. The synthesized materials exhibited high specific surface areas, while FTIR spectroscopy confirmed the successful incorporation of boron into the SnO_2_ matrix. XPS analysis indicated the presence of surface defects, including Sn^2+^ species and oxygen vacancies. Furthermore, the decrease in photoluminescence intensity with increasing boron content suggested a reduced electron–hole recombination rate. All investigated photocatalysts exhibited high photocatalytic activity toward crystal violet degradation under UV irradiation, 5 at% B-doped SnO_2_ sample, achieving more than 90% removal efficiency after 90 min. Scavenging experiments indicate superoxide radical and electrons as responsible for CV photodegradation. The enhanced photocatalytic performance is attributed to the synergistic effects of boron doping, including increased specific surface area, reduced charge-carrier recombination and defect-mediated charge transfer.

## Figures and Tables

**Table 1:** Structural parameters of synthesized samples.

Samples	2 Theta (°)			a = b ^1^ (Å)	c ^1^ (Å)	V_cell_ ^2^ (Å) ^3^	d ^3^ (nm)
	(110)	(101)	(211)				
SnO_2_	26.330	33.603	51.562	4.756	3.192	72.20	4.9
B1SnO_2_	26.327	33.616	51.576	4.776	3.184	72.63	3.7
B2SnO_2_	26.375	33.543	51.483	4.745	3.222	72.54	3.1
B5SnO_2_	26.300	33.581	51.614	4.757	3.188	72.14	3.0

^1^ a, b, c—lattice parameters, ^2^ V_cell_ is the cell volume, and ^3^ d (nm) is the average crystallite size.

**Table 2:** Textural parameters of SnO_2_ doped and undoped.

Sample	S_BET_ (m^2^/g)	S_micro_ (m^2^/g)	V_p_ ^1^ (cm^3^/g)	D_p_ ^2^ (nm)
SnO_2_	70.0	2.3	0.096	5.40
B1SnO_2_	75.8	19.1	0.055	3.97
B2SnO_2_	67.6	33.0	0.044	4.41
B5SnO_2_	88.1	32.2	0.065	4.34

^1^ Pore volume; ^2^ average pore diameter.

**Table 3:** The band gap values were calculated for both direct and indirect transitions.

Sample	E_d_ (eV)	E_i_ (eV)
SnO_2_	3.37	2.99
B1SnO_2_	3.35	2.92
B2SnO_2_	3.48	3.13
B5SnO_2_	3.41	3.01

**Table 4:** Comparative results of the removal efficiencies, band edge potentials, and kinetic parameters.

Sample	S_BET_	^1^ RE_ads_ (%)	^1^ RE_90_ (%)	^1^ RE_180_ (%)	^2^ E_CB_	^2^ E_VB_	^3^ k (min^−1^)	R^2^	^3^ t_1/2_ (min)
SnO_2_	70	63.0	86.4	89.4	0.05	3.42	0.01191	0.98946	58.2
B1SnO_2_	75.8	59.0	85.8	91.8	−0.01	3.48	0.01333	0.97626	52.0
B2SnO_2_	67.6	51.6	79.5	93.5	−0.01	3.56	0.01411	0.95322	49.1
B5SnO_2_	88.1	62.8	94.5	98.6	−0.03	3.51	0.01918	0.98724	36.1

^1^ RE_ads_, RE_90_, RE_180_—removal efficiencies after 30 min in the dark, 90 min. and 180 min, respectively, of UV irradiation; ^2^ E_CB_, E_VB_—conduction band edge and valence band edge potentials; ^3^ k, and t_1/2_—rate constant, and half-life (experimental conditions: 500 mg/L catalyst loading; CV initial concentration: 2 × 10^−5^ M).

## Data Availability

The original contributions presented in this study are included in the article/Supplementary Material. Further inquiries can be directed to the corresponding author.

## Supplementary Materials

The following supporting information can be downloaded at https://www.mdpi.com/article/10.3390/nano16171101/s1, Figure S1. XPS deconvolution for SnO_2_ sample of: (a) Sn3d. (b) O1s; Figure S2. XPS deconvolution for the B1SnO_2_ sample of (a) Sn3d. (b) O 1s; Figure S3. XPS deconvolution for the B2SnO_2_ sample of (a) Sn3d. (b) O1s; Figure S4. Tauc’s plot for determining direct band gap energies; Figure S5. Tauc’s plot for determining indirect band gap energies; Figure S6. Hydroxyl radical generation under simulated solar irradiation over TiO_2_ P25. Table S1a. XPS determined parameters for SnO_2_; Table S1b. XPS determined parameters for B1SnO_2_; Table S1c. XPS determined parameters for B2SnO_2_; Table S1d. XPS determined parameters for B5SnO_2_; Table S2. Comparison of the results for the photodegradation of Crystal Violet dye with the literature data.
