# Supplementary material for: Photocatalytic Activity of Boron-Modified SnO2 Nanoparticles for Crystal Violet Removal
Source: Nanomaterials (Basel). 2026 Sep 1;16(17):1101. doi: 10.3390/nano16171101 (PMC13567053; doi:10.3390/nano16171101)
Supplement: Supplementary file 1 [file nanomaterials-16-01101-s001.zip › nanomaterials-4442658-supplementary.pdf]

nanomaterials

Article

Photocatalytic activity of boron-modified SnO_2 nanoparticles for crystal violet removal

Daniela Negoescu[1], Anca Vasile[1], Oana Mocioiu[1], Crina Anastasescu[1], Mihaela Gherendi[2], Daniela C. Culita[1], Irina Atkinson[1], Simona Petrescu[1], Cristian Hornoiu[1], Veronica Bratan[1,*]

[1] Institute of Physical Chemistry – Ilie Murgulescu of the Romanian Academy, Bucharest, Romania; danielan@icf.ro (D.N.); avasile@icf.ro (A.V.); omocioiu@icf.ro (O.M.); canastasescu@icf.ro (C.A.); dculita@icf.ro (D.C.C.); iatkinson@icf.ro (I.A.); spetrescu@icf.ro (S.P.); chornoiu@icf.ro (C.H.)
[2] National Institute for Lasers, Plasma and Radiation Physics, 077125 Magurele-Ilfov, Romania; mihaela.gherendi@inflpr.ro (M.G.)
* Correspondence: vbratan@icf.ro

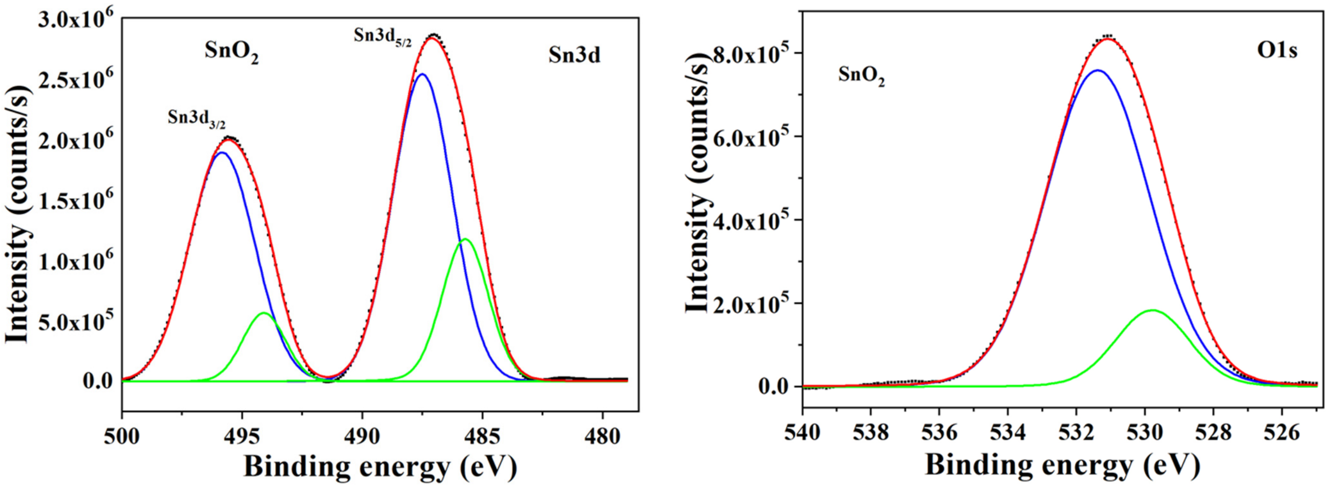

(a) (b)

Figure S1. XPS deconvolution for SnO_2 sample of: (**a**) Sn3d. (**b**) O1s.

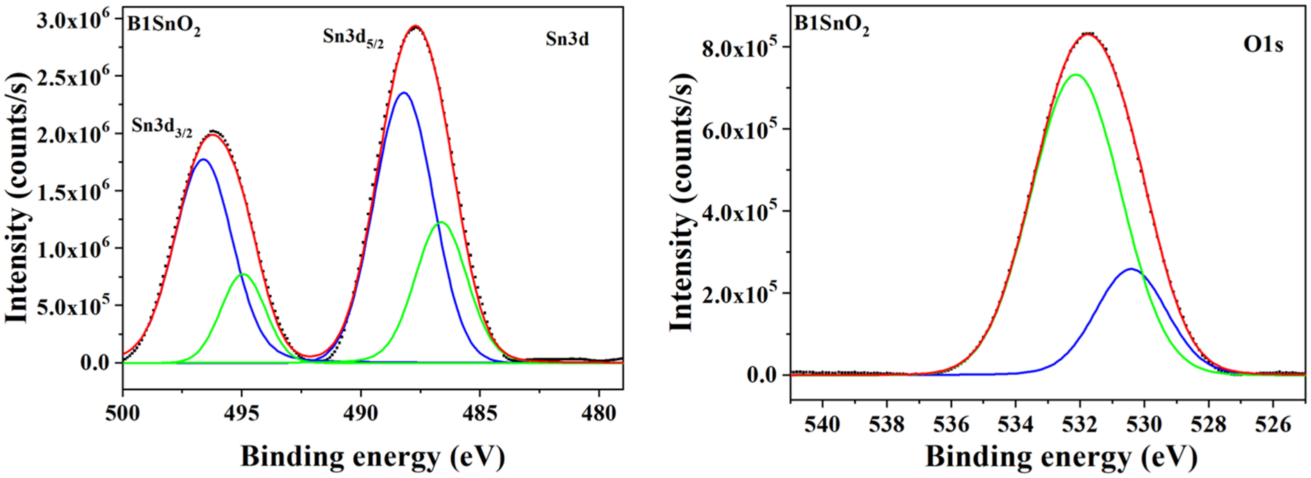

(**a**) (**b**)

Figure S2. XPS deconvolution for the B1SnO2 sample of: (**a**) Sn3d. (**b**) O1s.

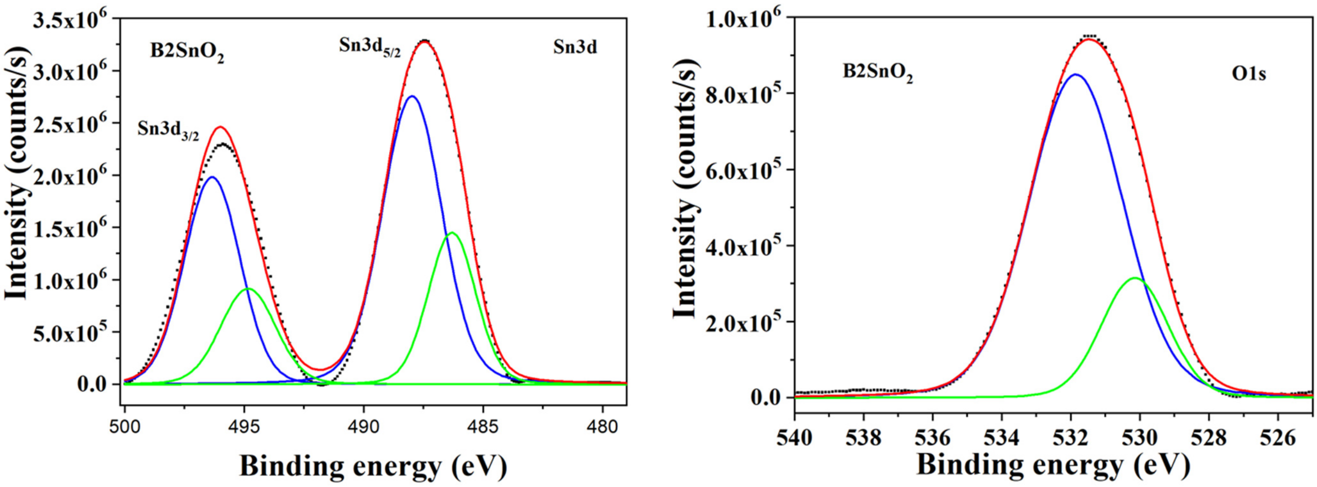

(**a**) (**b**)

Figure S3. XPS deconvolution for the B2SnO2 sample of: (**a**) Sn3d. (**b**) O1s.

Table S1a. XPS determined parameters for SnO_2

Peak	E_B (eV)	Composition (% at.)	Peak area (a.u)	Area ratio	ΔE (eV)	Obs.
Sn3d5/2	487.60	29.11	$7.08*10^6$	1.42	8.50	Sn^{4+}
Sn3d3/2	496.10		$5.10*10^6$			
Sn3d5/2	485.90		$3.8*10^6$	1.41	8.49	Sn^{2+}
Sn3d3/2	494.39		$2.86*10^6$			
O1s	531.39	70.89	$2.96*10^6$	5.7	1.59	O_v
O1s	529.8		$5.2*10^5$			O_{lat}

Table S1b. XPS determined parameters for B1SnO_2

Peak	E_B (eV)	Composition (% at.)	Peak area (a.u)	Area ratio	ΔE (eV)	Obs.
Sn3d5/2	488.16	29.11	8.30*10^6			
Sn3d3/2	496.63		5.73*10^6	1.45	8.47	Sn^{4+}
Sn3d5/2	486.33		3.15*10^6			
Sn3d3/2	494.86		2.11*10^6	1.49	8.53	Sn^{2+}
O1s	532.13	70.89	2.58*10^6			O_v
O1s	530.42		7.5*10^5	3.8	1.71	O_{lat}

Table S1c. XPS determined parameters for B2SnO_2

Peak	E_B (eV)	Composition (% at.)	Peak area (a.u)	Area ratio	ΔE (eV)	Obs.
Sn3d5/2	487.55	29.74	9.27*10^6			
Sn3d3/2	496.08		6.5*10^6	1.5	8.53	Sn^{4+}
Sn3d5/2	485.8		3.02*10^6			
Sn3d3/2	494.34		2.12*10^6	1.4	8.54	Sn^{2+}
O1s	531.53	70.26	2.73*10^6			O_v
O1s	529.88		5.7*10^5	4.8	1.65	O_{lat}

Table S1d. XPS determined parameters for B5SnO_2

Peak	E_B (eV)	Composition (% at.)	Peak area (a.u)	Area ratio	ΔE (eV)	Obs.
Sn3d5/2	488.01	28.9	8.42*10^6			
Sn3d3/2	496.45		5.86*10^6	1.46	8.44	Sn^{4+}
Sn3d5/2	486.23		4.66*10^6			
Sn3d3/2	494.68		3.08*10^6	1.51	8.45	Sn^{2+}
O1s	531.86	71.1	3.09*10^6			O_v
O1s	530.15		8.05*10^5	3.8	1.71	O_{lat}

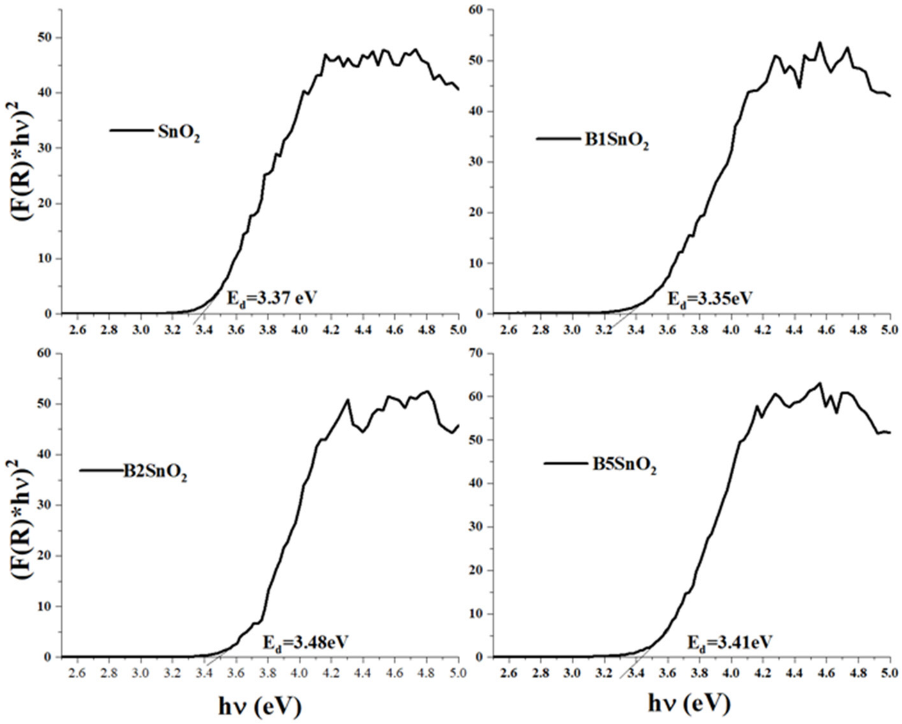

Figure S4. Tauc's plot for determining direct band gap energies

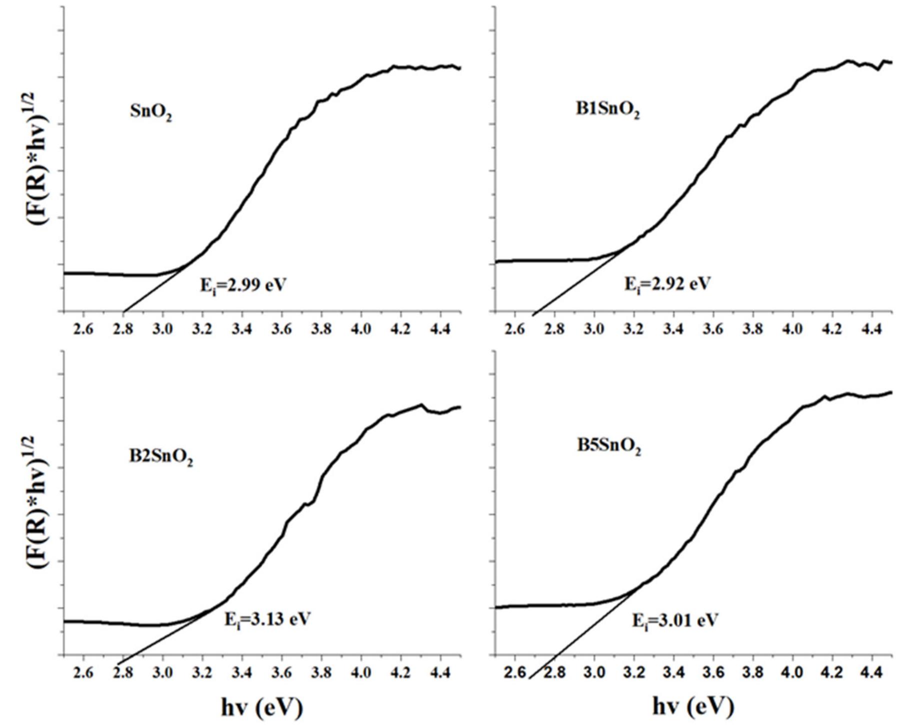

Figure S5. Tauc's plot for determining indirect band gap energies

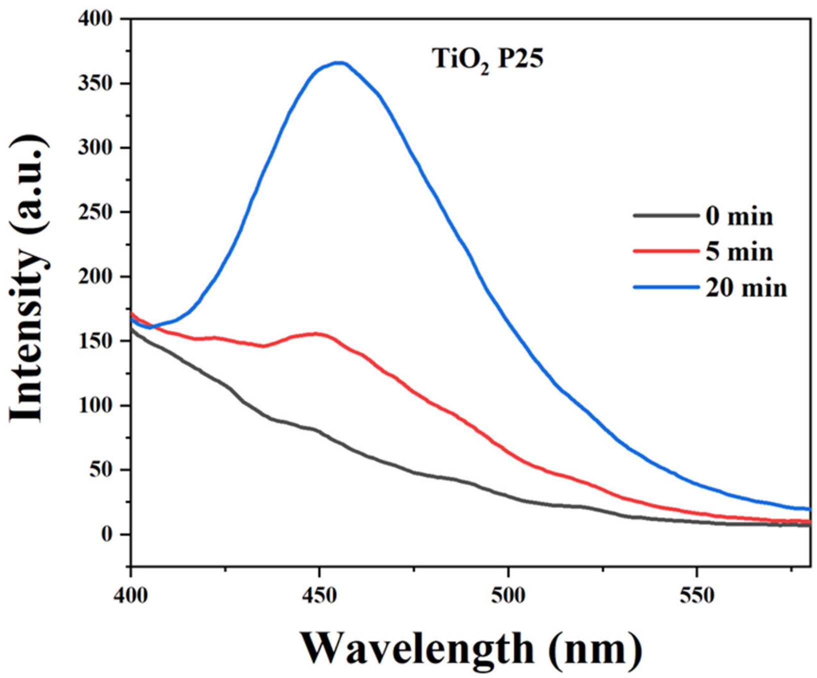

Figure S6. Hydroxyl radical generation under simulated solar irradiation over TiO_2 P25.

Table S2. Comparison of the results for the photodegradation of Crystal Violet dye with the literature data.

**Photocatalyst**	**Light Irradiation**	***Q (mg/L)**	***C mg/L)**	***T (min)**	***E %**	**Ref.**
TPB-Ag	UV	187.5	14	180	90.2	[6]
$AgTiO_2$	UV	1000	0.02	105	97	[66]
SnO_2	UV	1000	8.15	180	81	[67]
10NiO-ZnO	UV	100	100	180	100	[68]
ZnO	Vis	2500	14	60	70.6	[69]
$B5SnO_2$	UV	500	8.2	90	94.5	This study

* Q—catalyst concentration (mg/L); C—dye concentration (mg/L); T—time (min); E—efficiency of photodegradation (%).
